# Isolation, characterization, and annotation of two bacteriophage from North Carolina soil using *Arthrobacter globiformis* : HamCheese and Kihatsu

**DOI:** 10.17912/micropub.biology.001856

**Published:** 2026-01-02

**Authors:** Sheridan R Publico, Reagan R Rouland, Britnee M Bensinger, Wyatt W Clancy, Saskia M Hupertz, Ethan A Koeman, J. Trevor O'Neill, Neha Parthasarathy, Henry M Sederoff, Erik T Varela Hernandez, Reagan Cynthia, Stephanie L Mathews

**Affiliations:** 1 Department of Biological Sciences, North Carolina State University; 2 Department of Medicine, Nursing and Health Sciences, Monash University, Melbourne, Victoria, Australia; 3 College of Agricultural and Life Sciences , North Carolina State University; 4 Department of Chemical and Biomolecular Engineering, North Carolina State University; 5 Department of Forestry and Environmental Resources, North Carolina State University

## Abstract

Bacteriophage HamCheese and Kihatsu are siphoviruses isolated from soil in Raleigh, NC and Clayton, NC, respectively, using *Arthrobacter globiformis* , B2979. HamCheese is assigned to actinobacteriophage cluster AS based on gene content similarity. Its 38454 base pair genome encodes 67 putative genes. Kihatsu is assigned to cluster FF phage, with a genome 43,237 base pairs in length encoding 67 putative genes and 2 tRNAs. Based on gene content, both pages are predicted to be temperate.

**Figure 1. Plaque morphology of isolated phages:**
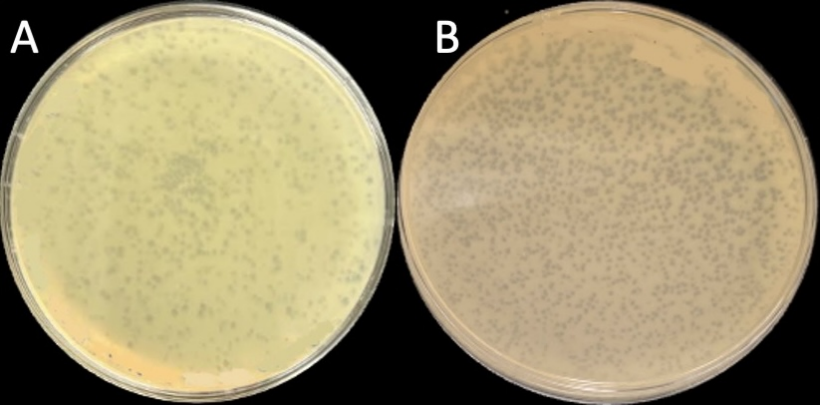
(A)HamCheese and (B)Kihatsu when plated on PYCa agar in 100 mm diameter plates with *A. globiformis* *B-2979* .

## Description

Bacteriophages (phages) are viruses that selectively infect and replicate in bacterial hosts. Isolation of new phages advances the current understanding of their diversity and phylogenetics and expands the collection of phages that can be examined for the therapeutic potential to treat bacterial infections (Hatfull et al. 2022). This study describes two bacteriophages that infect *Arthrobacter globiformis* B-2979, a bacterium commonly found in soil and part of the phylum Actinobacteria (Freise et al., 2025).

Bacteriophages HamCheese and Kihatsu were isolated using an enriched isolation method from soil samples collected in a grassy area beneath a birdfeeder in Raleigh, North Carolina (GPS 35.891502 N, 78.536568 W) and garden bed topsoil in Clayton, North Carolina respectively (GPS 35.689791 N, 78.424139 W). The enrichment was performed as described in Zorawik et al. (2024). Briefly, approximately 15 cm ^3^ of soil was suspended in 35 mL PYCa medium and shaken at 30°C for 1 hour followed by centrifugation at 2,000 g and then vacuum filtration (0.22 um filter) of the supernatant. This filtrate was inoculated with *A. globiformis* B2979 and incubated at 30°C for 5 days at 250 rpm. The filtered enrichment was then plated on PYCa agar with *A. globiformis* B-2979 and incubated at 30°C for 48 hours, producing small, turbid plaques 1 mm ± 0.05 in diameter (n=5) for HamCheese and 1 mm ± 0.09 in diameter (n=5) Kihatsu ( Figure 1A- B). HamCheese and Kihatsu were then purified by three successive rounds in which plaques were selected and plaque assays performed, after which lysates were prepared. Negative-staining transmission electron microscopy revealed both phages to have siphovirus morphology. HamCheese has a capsid diameter of 40 nm ± 0.5 and a tail length of 120 nm ± 10 (n=3) while Kihatsu has a capsid diameter of 62.5 nm ± 3 and a tail length of 237.5 nm ± 7 (n=3).

DNA was extracted from HamCheese and Kihatsu using the Promega Wizard DNA Cleanup Kit and prepared for sequencing with the NEB Ultra II FS Kit. The genomes were sequenced using an Illumina NextSeq 1000 (XLEAP-P1 kit), producing 2M and 1,381,149 100-base reads for HamCheese and Kihatsu, respectively. Raw reads were trimmed with cutadapt 4.7 (using the option: –nextseq-trim 30) and filtered with skewer 0.2.2 (using the options: -q 20 -Q 30 -n -l 50) prior to assembly (Martin 2011; Jiang et al. 2014; Wick et al. 2017; Gorden et al. 1998). Raw reads were assembled and checked for completeness using Newbler v2.9 with default parameters and Consed v29, respectively (Margulies et al., 2005; Gordon et al., 1998) *.* The genome of HamCheese assembled with 4,812x coverage, is 38,454 bp in length, with a 12 base 3' sticky overhang, 66% G+C content, and assigned to cluster AS3 based on gene content of at least 35% to phages in the Actinobacteriophage database phagesdb ( https://phageDB.org ) (Pope et al. 2017; Russell and Hatfull 2017). The genome of Kihatsu assembled with 1,823x coverage, is 43,184 bp in length, with a 12 base 3' sticky overhang, 64.9% G+C, and assigned to cluster FF, and was found to contain two tRNAs (Pope et al. 2017; Russell and Hatfull 2017).

The genome sequences were auto-annotated using DNAMaster v5.23.6 ( https://phagesdb.org/DNAMaster/ ) embedded with Genemark v2.5p (Besemer and Borodovsky, 2005) and Glimmer v3.02b (Delcher et al. 1999). Starterator was used to refine suggested start sites (Pacy 2016). Phage Evidence Collection and Annotation Network (PECAAN) v20221109 (Rinehart et al. 2016) was also used to analyze the phage genomes with Phamerator v519 using the "Actino_Draft" database version 627 (Cresawn et al. 2011), BLASTp (using the PhagesDB and NCBI nonredundant databases) (Altshcul et al., 1990), HHPred (using the PDB mmCIF70, Pfam-A, and NCBI Conserved Domain databases) (Zimmerman et al. 2018), and TMHMM v2.0 (Sonnhammer et al. 1998). Default settings were used for all software. Annotation of HamCheese resulted in 70 putative genes and 0 tRNA while Kihatsu has 68 putative genes with two tRNA identified by Aragorn v1.2.41.c (Laslett and Canback 2004) and tRNAscan-SE v2 (Lowe and Eddy 1997).

Both phages harbor tyrosine integrase and immunity repressor genes and are thus predicted to be temperate and consistent with lysogen formation observed for phages of cluster FF and AS3 that encode homologous integrase and immunity repressor gene products (Wise and Sivanathan, 2025; Jackson and Vega, 2025). Kihatsu contains two tyrosine integrase genes similar to other cluster FF phage such as QuinnAvery and Zaheer and it remains unclear if one or both are necessary for lysogen formation.

**Nucleotide sequence accession numbers**

HamCheese is available at GenBank with Accession No. PRJNA488469 and Sequence Read Archive (SRA) No. SRR32869143 . Kihatsu is available at GenBank with Accession No. PRJNA488469 and Sequence Read Archive (SRA) No. SRR32869158 .
